# Risk factors of migraine-related brain white matter hyperintensities: an investigation of 186 patients

**DOI:** 10.1007/s10194-011-0299-3

**Published:** 2011-02-18

**Authors:** Anita Trauninger, Eszter Leél-Őssy, David Olayinka Kamson, László Pótó, Mihály Aradi, Ferenc Kövér, Marianna Imre, Hedvig Komáromy, Szilvia Erdélyi-Botor, Ágnes Patzkó, Zoltán Pfund

**Affiliations:** 1 Department of Neurology, University of Pécs, 7623 Pécs, Rét u. 2, Pecs, Hungary; 2 Institute of Bioanalysis, University of Pécs, Pecs, Hungary; 3 Diagnostic Center, Pecs, Hungary

**Keywords:** Migraine, White matter hyperintensity, Disease duration and attack frequency, Stroke risk factors

## Abstract

Brain white matter hyperintensities are more prevalent in migraine patients than in the general population, but the pathogenesis and the risk factors of these hyperintensities are not fully elucidated. The authors analyzed the routine clinical data of 186 migraine patients who were referred to the Outpatient Headache Department of the Department of Neurology, Medical School, University of Pécs, Hungary between 2007 and 2009: 58 patients with white matter hyperintensities and 128 patients without white matter hyperintensities on 3 T MRI. Significant associations between the presence of white matter hyperintensities and longer disease duration (14.4 vs. 19.9 years, *p* = 0.004), higher headache frequency (4.1 vs. 5.5 attacks/month, *p* = 0.017), hyperhomocysteinemia (incidence of hyperintensity is 9/9 = 100%, *p* = 0.009) and thyroid gland dysfunction (incidence of hyperintensity is 8/14 = 57.1%, *p* = 0.038) were found. These data support the theory that both the disease duration and the attack frequency have a key role in the formation of migraine-related brain white matter hyperintensities, but the effects of comorbid diseases may also contribute to the development of the hyperintensities.

## Introduction

Migraine is a complex disorder of the brain, and its pathomechanism is intensively investigated. Migraine is not as benign a disease as it was thought before the imaging era since migraine is an independent risk factor for deep white matter lesions, silent posterior circulation territory infarcts, and infratentorial hyperintense lesions [1–4]. Migraine patients have an almost fourfold greater risk to develop white matter lesions than non-migraine controls [5], and the prevalence of these lesions is higher in migraine with aura than in migraine without aura [4]. The risk of developing white matter lesions is higher in female migraineurs and in patients with higher attack frequency and longer disease duration [4, 6]. Furthermore, it seems that brain white matter lesions do not correlate with age [7, 8]. It is also known that beyond the existing relation between migraine and stroke, there is also an association between migraine and coronary heart disease [9, 10]. Since the risk of these severe complications is low in the general population, it is not possible to identify which migraine patients will develop a cerebrovascular or cardiovascular event [10].

The clinical significance of white matter hyperintensities (WMH) is uncertain and the pathogenesis of these lesions is likely multifactorial. A number of pathophysiological mechanisms have been proposed including attack-related oligemia and focal hypoperfusion [3, 11], glutamatergic excitotoxicity [11, 12], immune-based white matter demyelination [13], and mitochondrial dysfunction [14]. Several studies considered the role of inflammation as a vascular risk for brain white matter lesions in migraine [15–17]. Elevated C-reactive protein found in migraine patients is a sensitive indicator of active systemic inflammation and a marker of oxidative stress [15]. Increased proinflammatory cytokines (IL-1, IL-6, TNF-α) have been reported during acute migraine attacks, as well as in the interictal periods [16, 17]. Repeated sterile vascular inflammation results in endothelial injury of the cranial blood vessels, cranial arteriopathy, and consequent thrombosis [18, 19]. It has been suggested that cortical spreading depression causes disruption of the blood brain barrier through a matrix metalloproteinase-9-dependent cascade mechanism which may result in local tissue damage [20]. It is also known that endothelial dysfunction associates with an increased rate of cerebrovascular ischemic events, and genetic factors (such as angiotensin-converting enzyme insertion/deletion, methylenetetrahydrofolate reductase C677T polymorphisms, and von Willebrand factor activity) which increase the susceptibility of endothelial dysfunction have been linked to migraine [21, 22].

Although migraineurs, particularly with aura, have a higher cardiovascular risk profile than individuals without migraine [23], the presence of white matter lesions proved to be independent of a history of hypertension, diabetes, smoking, hypercholesterolemia, hyperhomocysteinemia, patent foramen ovale with right-to-left shunt, and oral contraceptive use [5, 24, 25]. Associations with antiphospholipid antibodies (anticardiolipin antibodies, lupus anticoagulans) and abnormal coagulation parameters (antithrombin-III, protein S, protein C) were also not found [26].

Because of the high prevalence of white matter hyperintensities, and the partly contradictory and uncertain etiological data, we have investigated the possible risk factors for subclinical brain white matter hyperintensities in our own migraine patients by analyzing their routine clinical data.

## Patients and methods

### Subjects and studies

Subjects included in this study were referred to the Outpatient Headache Department of the Department of Neurology, Medical School, University of Pécs, Hungary between 2007 and 2009. All the patients who were chosen for the study met the criteria of migraine patients as defined by the International Headache Society [27]. Further, patients were also selected based on the lack of major comorbidities, such as hypertension, cardiac disease, diabetes, thyroid gland dysfunction, oncological and haematological diseases, infectious diseases (e.g. HIV, hepatitis), central nervous system demyelination (e.g. multiple sclerosis), and genetically inherited disorders (e.g. CADASIL). Altogether 186 migraine patients were investigated in the study (141 patients without aura, 45 patients with aura, age range 18–58 years, mean age 36.4 years, SD: 8.9; 156 females and 30 males). Based on the findings of brain magnetic resonance imaging (MRI) studies, patients were divided into two groups: patients with WMH (WMH+ group, *n* = 58, mean age 40.2 year, 54 patients had supratentorial hyperintensities, 4 patients had cerebellar hyperintensities) and patients without WMH (WMH− group, *n* = 128, mean age 34.9 year).

All participants underwent a structured clinical examination (history taking, physical examination, blood pressure measurement, serum and urine tests, brain MRI study) to identify comorbid medical disorders. Gender, migraine type, disease duration, attack frequency, history of smoking and taking oral contraceptives, serum cholesterol (reference range 4.00–5.60 mmol/l) and low-density lipoprotein (LDL) (reference range 0.00–3.40 mmol/l), uric acid (reference range 180–350 μmol/l), homocysteine levels (reference range 5.00–15.00 μmol/l), and thyroid-stimulating hormone levels (reference range 0.27–4.20 mU/l) with thyroxine and triiodothyronine when necessary were routinely examined. Subclinical hypo- and hyperthyroidism was diagnosed when peripheral thyroid hormone levels were within normal reference laboratory range but serum thyroid-stimulating hormone levels were mildly elevated or decreased. In all cases when aura symptoms and/or WMH were present, patients were tested for vasculitis (antinuclear antibody, antineutrophil cytoplasmic autoantibody, antiphospholipid antibody, lupus anticoagulant), for Lyme disease (serum ELISA screening with Western blot testing), and for patent foramen ovale (transthoracic and transesophageal echocardiography).

### MRI protocol

The brain MRI examinations were carried out with an MR scanner operating at 3 T (Siemens Trio Tim, 12 channel head coil). A qualified neuroradiologist who was blinded to migraine diagnosis and clinical data rated the WMH. WMH were considered if visible as hyperintense on T2-weighted and FLAIR images, without hypointensity on T1-weighted scans, and were larger than 3 mm [28].

### Statistical analysis

Statistical analysis was performed using the SPSS 15.0 statistical package (SPSS Inc., Chicago, IL, USA). Chi-square tests were performed to assess the difference between the WMH+ and WMH− groups in relation to migraine type, disease duration, attack frequency, gender, smoking, cholesterol, LDL, uric acid, and thyroid-stimulating hormone levels. To examine the disease duration, patient groups were created in which the disease duration increased by 5 years in each group (0–5, 5–10, etc.). Furthermore, patients were divided into three groups by monthly attack frequency (rare: 0–1 attacks/month, average: 2–7 attacks/month, very frequent: 8 or more attacks/month). Since homocysteine level was measured only in 60 patients, and thyroid gland dysfunction was found only in 14 patients, a Fisher’s exact test was used to assess the difference between the groups. Both the disease duration and attack frequency were used as dichotomous variables when contingency tables were created and evaluated. Chi-square tests and Fisher’s exact test were used for these evaluations. The non-parametric Mann–Whitney test was also applied to investigate the role of the disease duration and the attack frequency in the formation of WMH in different groups, including all patients and migraine subgroups (patients without and with aura). In this analysis, the disease duration and the attack frequency were analyzed as continuous variables. Finally, a binary logistic regression was also performed to predict the presence of WMH from a set of independent variables. For this analysis homocysteine was not used due the number of the tested patients. Group comparisons were not performed for oral contraceptives because most of the migraineurs were female and there was contraceptive use in the past and/or current medical history in almost all cases. A value of *p* < 0.05 was considered to be significant.

## Results

1. There was no statistical difference between females (*n* = 48 out of 156, 30.7%) and males (*n* = 10 out of 30, 33.3%), as well as between migraine patients without aura (*n* = 42 out of 141, 30.5%) and patients with aura (*n* = 16 out of 45, 35.6%) in relation to the presence of WMH.
2. The number of patients with WMH increased with the increase of disease duration; in patients with above 20 migraine years hyperintensities were significantly more frequent than in those with less than 20 years of duration (under 20 years *n* = 28/120, 23.3%, above 20 years in *n* = 30/66, 45.6%, *p* = 0.007). When examining the association between the disease duration and the presence of WMH according to migraine type, it was found that both migraine patients without and with aura have a longer disease duration with WMH, than patients without WMH (Table 1).

**Table 1:** Differences between the WMH− and WMH+ groups in relation to disease duration and attack frequency in migraine patients without and with aura

	Patient number	Disease duration (years)	*p* value	Attack frequency (attack/month)	*p* value
All patients					
WMH−	128	14.3 ± 8.5	0.004	4.1 ± 3.0	0.017
WMH+	58	19.9 ± 11.6		5.5 ± 3.6	
Patients without aura					
WMH−	99	14.7 ± 8.7	0.030	4.6 ± 3.1	0.016
WMH+	42	19.7 ± 11.7		6.2 ± 3.6	
Patients with aura					
WMH−	29	13.1 ± 7.9	0.047	2.4 ± 2.4	0.067
WMH+	16	20.3 ± 11.8		3.5 ± 3.0	

*WMH−* patients without white matter hyperintensities, *WMH+* patients with white matter hyperintensities

The diseases duration and attack frequency values are presented in mean ± 1 SD

*p* values are based on Mann–Whitney statistical analysis
3. Although the proportion of migraine patients with WMH increased with the monthly migraine attack frequency (0–1 attack/month: *n* = 7/37, 18.9%, 2–7 attacks/month: *n* = 36/114, 31.5%, ≥8 attacks/month: *n* = 15/35, 42.9%), there was only a trend towards statistical significance (*p* = 0.08**)** when the patients were examined in three groups according to their attack frequency. Conversely, when the attack frequency of the WMH+ group was compared to the attack frequency of the WMH− group, a significantly higher attack number was found in the WMH+ group (Table 1). When examining the effect of attack frequency on the presence of brain WMH in migraine subgroups, it was found that migraine patients without aura and with WMH have a higher attack frequency than patients without aura and without WMH (Table 1). In patients with aura the same tendency was seen, but the difference was not statistically significant (Table 1).
4. WMH did not occur more frequently in smokers (*n* = 18/52, 34.6%) than in non-smokers (*n* = 40/134, 29.8%), but smoking was significantly associated (*p* = 0.001) with increased monthly attack frequency (<5 attacks/month: *n* = 87/134, 64.9% in non-smokers, *n* = 47/134, 35.0% in smokers, ≥5 attacks/month: *n* = 20/52, 38.4% in non-smokers, *n* = 32/52, 61.5% in smokers).
5. There was a significant relation between abnormally high serum homocysteine levels and the incidence of brain WMH; out of 60 patients tested for serum homocysteine 9 had WMH and homocysteine levels were elevated in all 9 patients (*p* = 0.009).
6. Subclinical hypo- (*n* = 8) and hyperthyroidism (*n* = 6) was detected in 14 patients (14/186 = 7.5%). Out of these patients, eight had WMH (4 patients in both groups, 8/14 = 57.1%). Statistical analysis showed the subclinical thyroid dysfunction occurred significantly more frequently in the WMH+ group than in the WMH− group (*p* = 0.038).
7. Although the statistical analysis did not show an increased risk of WMH in migraineurs with high cholesterol + LDL cholesterol (*p* = 0.06) and high uric acid levels (*p* = 0.07), the cholesterol + LDL cholesterol and uric acid values were found more frequently in the elevated range in the WMH+ group (*n* = 18/41, 43.9% for cholesterol + LDL cholesterol, *n* = 7/15, 46.6% for uric acid) than in the WMH− group (*n* = 40/145, 27.5% for cholesterol + LDL cholesterol, *n* = 51/171, 29.8% for uric acid).
8. When the effects of all the predictor variables were examined, only two good predictor variables were found: the disease duration (*p* < 0.01) and the attack frequency (*p* < 0.05) (Table 2). There was no significant effect with all the other variables (*p* > 0.1) (Table 2).

**Table 2:** Results of binary logistic regression for white matter hyperintensities

Examined variables	*p*	OR	95.0% CI for OR	
			Lower	Upper
Step 1				
Disease duration	0.090	1.046	0.993	1.103
Attack frequency	0.018	1.164	1.026	1.321
Age	0.913	0.997	0.947	1.049
Gender	0.865	1.087	0.414	2.855
Smoking	0.249	0.584	0.234	1.457
Thyroid function	0.289	0.637	0.277	1.465
Cholesterol	0.135	0.498	0.200	1.242
Uric acid	0.447	0.515	0.093	2.849
Step 7				
Disease duration	0.005	1.051	1.015	1.089
Attack frequency	0.017	1.153	1.026	1.295

The table presents the *p* value, the odds ratio (OR) and the 95% confidence interval (CI) for the OR for all of the variables at Step 1 and for the variables that were significant at Step 7 of the backward stepwise elimination procedure
9. An elevated antiphospholipid antibody titer was only found in one patient. Echocardiography showed a tiny patent foramen ovale with right to left shunt in two migraine patients with aura and with brain WMH. There was no patient with pathologic Lyme serology.

## Discussion

In this study, we have investigated possible risk factors for brain WMH based on migraine history and blood tests. To get more accurate data on those factors which may influence the formation of WMH, diseases which can associate with the presence of brain WMH without migraine, including hypertension, were excluded from our study. We found significantly higher hyperintensity incidence in patients with longer migraine duration, higher headache frequency, subclinical hyper- and hypothyroidism, and elevated plasma concentrations of homocysteine, while in the cases of cholesterol and uric acid, there was a trend towards statistical significance. Statistical difference was not found between migraine patients without and with aura in relation to the presence of WMH, but most of the patients in the WMH+ group had only supratentorial signal abnormalities [1]. There are differences between our results and the previously documented ones, and we suppose that these are based on the limitations of the performed studies due to differences in patient selection and sample size.

The relation between disease duration and attack frequency to the WMH is not surprising if we take into account the pathophysiology of migraine [29]. During the attack several intracranial pathologic processes are detectable, including intracerebral haemodynamic changes, local inflammatory responses, excessive neuronal activation and excitotoxicity, which may all lead to tissue damage [30]. Although there are regional differences and predilection sites for tissue damage, basically, the migraine attack affects the whole brain [6, 30, 31]. It is known that there are differences among migraine patients in relation to the risk of the WMH. The risk depends not only on the disease duration and frequency, but the migraine type, attack duration, and comorbid conditions can also influence it [4, 6]. The comorbid conditions are mainly routine stroke risk factors and these may lead to tissue damage by direct (e.g. blood vessel endothelium dysfunction, hypercoagulation, embolization) or indirect (e.g. smoking) effects [21, 23, 32–41]. In our study, the occurrence of WMH was not higher in smokers than in non-smokers, but smoking increased the headache frequency, therefore smoking may indirectly cause white matter hyperintensities. These data are consistent with the previously reported ones showing higher prevalence rates for headache amongst smokers compared to non-smokers [32, 33].

Plasma homocysteine concentration is controlled by genetic (MTHFR C677T mutation), nutritional (vitamins folate, B6, B12) and acquired (smoking, alcohol consumption, renal diseases, malignancies, inflammation, daily physical activity) factors [34]. It is also known that endothelial asymmetric dimethylarginine has been linked with elevated levels of homocysteine [35]. Conversely, asymmetric dimethylarginine concentrations are substantially elevated by native or oxidized LDL cholesterol [36]. Furthermore, HDL-cholesterol levels were inversely correlated to the homocysteine levels [37]. In this study, we did not examine which factors could lead to an elevation of homocysteine levels, only if the elevation itself was present. Regardless of the provoking factors, elevated total homocysteine concentration is an independent risk factor for recurrent stroke [38] which may result from an endothelial injury and altered coagulant properties of the blood [39, 40]; however, it is still controversial whether mild hyperhomocysteinemia is a causal factor. It has been found that migraine patients with aura who are homozygotes for methylene-tetrahydrofolate reductase (MTHFR) C677T variant, are at risk for elevated levels of homocysteine, and homocysteine-related endothelial dysfunction may be involved in the initiation and maintenance of migraine [21].

Although subclinical thyroid gland dysfunction was found in few patients in our study, more than half of these patients had brain WMH. Subclinical thyroid gland dysfunction can be a risk factor for WMH in migraine, but currently there is no definite evidence to confirm this. Previously, in a large cross-sectional population-based study, TSH levels were lower among headache patients, especially migraineurs, than in those without headache complaints [41]. It is also known that the endocrine function, including the plasma concentrations of TSH, is altered in chronic migraine patients with high headache frequency and frequent analgesic use [42]. In addition, a significant correlation was found between the duration of the disease and the altered hormonal response [42]. Hyperthyroidism can associate with atrial fibrillation and cardioembolic stroke and may lead to a hypercoagulability state [43]. Hypothyroidism is associated with a worse cardiovascular risk factor profile including elevated cholesterol and low-density lipoprotein levels, diastolic hypertension, increased homocysteine and C-reactive protein concentrations, impaired thyroid hormone action on target tissue by smoking, tendency toward decreased fibrinolytic activity in mild and moderate hypothyroidism, and endothelial dysfunction with progression to atherosclerosis [43].

We found WMH more frequently in our migraine patients with elevated serum cholesterol and uric acid levels, and we speculate that if there is a relation between migraine and cholesterol and uric acids levels, this can be based on altered endothelial dysfunction and migraine attack frequency. Hyperlipoproteinemias can be familial in origin but there are drugs and diseases (e.g. oral contraceptives, hypothyroidism) which can cause secondary hyperlipoproteinemias or worsen underlying hyperlipoproteinemic states [44]. High levels of blood lipids and free fatty acids are among the factors involved in triggering migraine headache [45]. The presence of hypercholesterolemia and dyslipidemia in patients with migraine may increase the risk of vascular wall injury. Hypercholesterolemia is associated with an increase in endothelial permeability, the retention of lipoproteins within the intima of blood vessels, inflammatory cell recruitment and foam cell formation filled with oxidative-LDLs, and finally these processes progress to atherosclerotic plaque maturation [46]. Cholesterol crystals, a component of human atherosclerotic plaques, could also cause an inflammatory response and neuronal injury in the brain with persistent activation of microglia and astrocytes via microembolization [47]. Furthermore, a number of epidemiologic data show that an elevated serum uric acid level is a powerful predictor of an increased risk of a cardiovascular event including stroke and silent brain infarcts [48] however, the available data are contradictory. It is entirely plausible that chronic elevations in serum uric acid levels have harmful effects on platelet, smooth muscle, and endothelial function [49], but the neuroprotective, antioxidant effect of high uric acid levels may associate with improved outcome in the peri-ictal period [50].

In conclusion, this study provides additional data on the etiology of migraine-related WMH supporting the former assumptions that a wide range of factors contribute to lesion formation. These include attack-related intracerebral changes and direct or indirect effects of comorbid diseases. Development in understanding the pathophysiology of migraine and the pathology of WMH, the use of effective therapy for migraine attack and prophylaxis, detection and treatment of vascular risk factors, and avoiding smoking may help to prevent the development of the hyperintensities.
